# Negotiating organisational blame to foster learning: Professionals’ perspectives about Domestic Homicide Reviews

**DOI:** 10.1111/hsc.13725

**Published:** 2022-01-20

**Authors:** Alina Haines‐Delmont, Kelly Bracewell, Khatidja Chantler

**Affiliations:** ^1^ Department of Nursing Manchester Metropolitan University Manchester UK; ^2^ 6723 School of Social Work, Care and Community University of Central Lancashire Preston UK

**Keywords:** domestic homicide reviews, domestic homicides, domestic violence and abuse, family involvement, organisational blame, recommendations

## Abstract

Domestic Homicide Reviews (DHRs) are a statutory requirement in England and Wales, conducted when somebody aged 16 and over dies from violence, abuse or neglect by a relative, intimate partner or member of the same household. While key aims of DHRs are to identify recommendations and lessons learned to eventually prevent further domestic homicides, there is limited evidence globally regarding the extent to which these are followed up or make a difference. This paper explores the barriers and facilitators to the conduct and impact of DHRs to enhance their learning potential. It is based on nineteen qualitative interviews with professionals involved in the DHR process across five Safeguarding Boards in Wales and fourteen Community Safety Partnerships in the North‐West of England, UK. Findings are presented thematically under four section headings: upskilling and democratising the review process; family and friends’ involvement; negotiating organisational blame to foster learning; and actioning and auditing recommendations. It is suggested that organisational learning cannot be achieved without accepting organisational responsibility, which could be interpreted as blame. The role and skills of the Chair are perceived as key to ensure a safe, evidence‐based, transparent and learning‐focused DHR process. Developing and actioning recommendations may challenge longstanding prejudices. Promoting the role of families/survivor networks and professionals on an equal footing would support a more democratic process. Learning could be enhanced by thematising recommendations and proactively using lessons from one area to inform another. Participants called for appropriate central regulation and accountability to support the action of recommendations.

**What is known about this topic?**

- Domestic Homicide Reviews (DHRs) (or their equivalents) aim to improve support to domestic abuse victims to prevent domestic homicides.
- DHRs aim to foster a ‘learning’ rather than a ‘blame’ culture.
- Little is known globally about whether recommendations included in DHRs are implemented.

**What this paper adds**

- DHRs involve a complex process where the facilitative skills of the Chair, meaningful involvement of families and survivor networks and openness to learning are key.
- To maximise learning and action from DHR findings, participants called for a greater role for national bodies (e.g. the Home Office).
- Disrupting the hierarchy between statutory organisations and the voluntary sector enriches the DHR process.

## 1 INTRODUCTION

Domestic violence and abuse (DVA) is a significant human rights and gender‐specific global issue. At least one‐third of women have experienced physical and/or sexual violence by an intimate partner (United Nations Statistics Division, 2021; World Health Organization, 2013, 2021). While the majority of homicide victims are male, most victims of intimate partner/family‐related homicide are women (UN Women, 2019; United Nations Office on Drugs & Crime, 2018). DVA is defined as ‘any incident or pattern of incidents of controlling, coercive, threatening behaviour, violence or abuse between those aged 16 or over who are, or have been, intimate partners or family members regardless of gender or sexuality. The abuse can encompass, but is not limited to psychological, physical, sexual, financial, emotional’ (Home Office, 2018). The Domestic Abuse Act (HM Government, 2021) puts this definition on a statutory footing.

Domestic Homicide Reviews (DHRs) were legally mandated in England and Wales in 2011 to understand the antecedents to homicide including service responses and identify missed opportunities for intervention (HM Government, 2004; Home Office, 2016). This represents a significant shift from a blame and culprit‐centred legalistic process to one focused on identifying system and environmental factors to enable learning. Various terms are utilised in different countries including Domestic Violence Death Review Committee (Canada) and Domestic Violence Fatality Reviews (US). This article utilises the term DHR as the research was conducted in England and Wales where DHR is the appropriate terminology. Despite different structures, funding systems, case selection, definitions of domestic homicide and make‐up of committee members, they have the common aim of learning from domestic homicide to strengthen policy and practice to prevent such deaths.

In England and Wales there are often parallel reviews including serious case reviews, mental health reviews and adult practice or safeguarding adult reviews. As these reviews utilise significant resources, questions are raised regarding their investigative approach and effectiveness (Holliday & Taylor, 2015). Robinson et al. (2019) argue for streamlining the review processes to reduce duplication given their overlaps and to enable meaningful and lasting change.

The extent to which learning gained from DHRs has informed policy and practice regarding DVA is unclear. Many reports produce broadly similar recommendations which may indicate limited wider impact or that some recommendations are needed on a long‐term basis (e.g. training) or require substantial systemic change.

International literature demonstrates that reviews are complex processes and that the composition and roles of panel members influence recommendations and learning (Bent‐Goodley, 2013). Albright et al. (2013) draw attention to the potential for defensiveness within panels; Websdale (2003) highlights the need for adequate funding; others argue for greater panel diversity to ensure a more holistic approach (Albright et al., 2013; Marsh Pow et al., 2015); Websdale et al. (1999) stress the importance of learning rather than apportioning blame to ensure that key messages can be harnessed to facilitate change; while Mullane (2017) highlights the need to focus on the victim through the involvement of family and friends.

## 2 AIMS AND OBJECTIVES

The main aim of the study was to explore professionals’ experience and views about the DHR process to better understand the opportunities and challenges presented by DHRs. Specific objectives included:

- Exploring professionals’ experience of panel membership or chairing; views on commissioning of DHRs, timeframe, writing and publication of the report;
- Exploring views about the role of families and friends, benefits and limitations;
- Capture views and knowledge about implementation of recommendations; and
- Identify key areas for improving practice.

## 3 METHODS

This paper reports on findings from a larger domestic homicide research study funded by the UK Economic and Social Research Council (ESRC). Nineteen qualitative semi‐structured interviews were conducted between June 2020 and March 2021. Participants were recruited using a convenience sampling strategy targeting professionals with DHR experience from four Safeguarding Boards in Wales and fourteen Community Safety Partnerships (CSPs) in North‐West England with DHR experience. Ethical approval for the study was obtained from Manchester Metropolitan University's Health, Psychology and Social Care Research Ethics and Governance Committee on 07/02/2020 (EthOS Reference Number: 20152). Subject to written informed consent, participants were interviewed remotely by three researchers. Participants had access to the interview topics and understood the sensitivity of the research and any potential risks, prior to consent to participate. While the interviews were conducted in a way to minimise distress, participants were provided with contact information if they needed additional support.

Key questions were used (based on the team's prior work, existing evidence and consultation with the study's advisory committee) but each interview was flexible and driven by the discussion and/or the role of the participant. Interviews were audio recorded, transcribed verbatim, and anonymised. Transcripts were uploaded on NVivo v.2020 (QSR International, 2020) for reading, coding and analysis.

Two authors analysed the data thematically to identify patterns of meaning across the dataset (Braun & Clarke, 2006). Data were analysed both inductively and deductively and analysis was reflective and iterative. A robust set of codes was developed followed by an iterative process of theme development and refinement. Most codes were clustered into ‘higher level’ patterns to identify candidate themes. These were then discussed with the principal investigator and the study's advisory committee to ensure the thematic analysis addressed the study objectives. Finally, consensus meetings with the principal investigator were held to agree on the emerging themes and develop analytic narratives for each theme.

Anonymised excerpts from interviews are used to illustrate the points being made. Each participant was given a code (e.g. ‘Professional 1, Wales’; ‘Professional 1, England’). Reference to participants’ gender, age, qualification, role and organisation was removed from the data to preserve anonymity.

Participants were from health services, local authority, the government, police, probation and offender rehabilitation, charity/third sector specialists; within CSPs or Safeguarding Boards and experience of involvement in the DHR process.

## 4 FINDINGS

**FIGURE 1:**
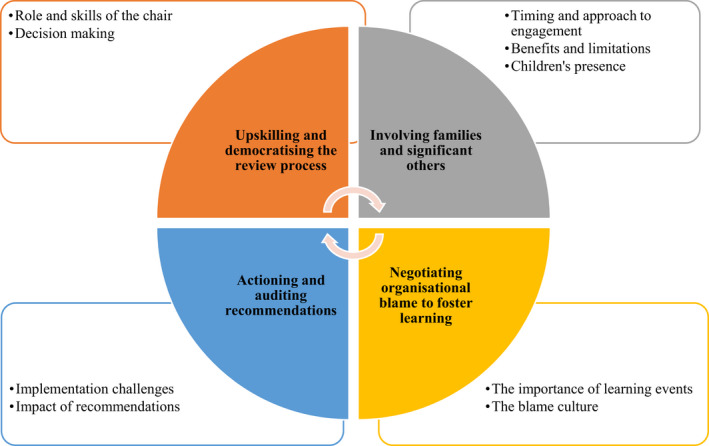
Emerging main themes and sub‐themes

The four main themes identified are (a) upskilling and democratising the review process; (b) family and friends’ involvement; (c) negotiating organisational blame to foster learning; (d) actioning and auditing recommendations. Themes and sub‐themes are illustrated in Figure 1 below. Each theme is discussed in turn.

### 4.1 UPSKILLING AND DEMOCRATISING THE REVIEW PROCESS

#### 4.1.1 Role and skills of the Chair

The Chair's skills, experience, and working style were viewed as important enablers or limiters to all aspects of the DHR process. Participants perceived good Chairs as enabling learning, engendering honesty and asking difficult and pertinent questions. They empowered professionals to be objective, question practice, challenge entrenched attitudes and prejudice. The ideal Chair was seen as skilled in dealing with sensitive and contentious issues, so those involved in the DHR process could feel safe to talk openly or feel listened to in a non‐judgemental way:

[…] agencies would get very protective over their actions and so you need a very strong Chair to be able to ask those difficult questions, to make people maybe just look at their own practice without it being a finger pointing. (Professional 1, England)

Some of the Chairs that we have worked with are exceptional […] the sign of a really good Chair if they are able to get that information out of agencies without putting them on the defensive. (Professional 2, England)

Employing an independent Chair was seen as advantageous, but was counterbalanced by funding issues and insufficient knowledge of the local area. The disappointment with poor quality reviews, especially those rejected by the Home Office, was evident, particularly when Chairs failed to question their own assumptions which sometimes contributed to victim‐blaming, compromised objectivity and lack of professionalism:

I think on the one occasion, the Chair said to us, “He was very good to her. He supported her financially and provided her with a home, this, that and the other.” I said, “Yes, he was lovely to her up until the day he killed her.” So he almost blamed the victim. (Professional 8, Wales)

#### 4.1.2 Decision making

According to some participants, the DHR review process is not entirely democratic. First, it is perceived as limited by a lack of diversity and valuable input from third sector specialist DVA agencies:

So, you get vulnerable people that are perhaps more honest with the third sector organisations, but I think that is a bit of ongoing historic snobbery, is not it, that statutory sectors know best. So, I think the panels need to be representative and balanced really. (Professional 3, Wales)

Second, decision‐making is heavily influenced by a Chair who may not always consider the expertise of the panel members:

[…] it is been quite frustrating that you do not seem to have the same influence and it does not seem to be a collaborative approach. In another review locally we had a different Chair but equally we had problems in that they wanted recommendations that we as [name of agency] knew were not achievable. (Professional 1, Wales)

Despite Home Office guidance as to when reviews should be conducted, participants highlighted grey areas, especially for cases where there was no agency involvement prior to the homicide. They argued that all cases need to be carefully considered to ensure that decision‐making regarding whether to conduct a DHR was robust. However, not commissioning a DHR, raises questions about the impact on the family:

Because they were not known to agencies, it does not mean that the family then do not get to have their story and share their views of that. (Professional 11, Wales)

Significantly, democratising the DHR process is about meaningful involvement of families and significant others. Centring the victim's voice via families is a key rationale for DHRs, yet this is a complex process as discussed next.

### 4.2 INVOLVING FAMILIES AND SIGNIFICANT OTHERS

#### 4.2.1 Timing and approach to engagement

Professionals recognised the increasing importance of involving friends and relatives, particularly since the updated Home Office guidance in 2016. They described feeling cautious about approaching or managing expectations. Timing is key and requires sensitivity:

In the one case […] we were too late, in the other one because I think we were too early […] People do need time to be able to get over one aspect of the grieving process in order to be able to move on and to do something about it. (Professional 9, Wales)

Professionals suggested that families might need to be approached more than once, as levels of involvement vary and views on participation can change over time. Early involvement of specialist support organisations such as the National Homicide Service or Advocacy After Fatal Domestic Abuse (AAFDA) was recommended.

Participation of families was managed at the Chair's discretion.

#### 4.2.2 Benefits and limitations

The involvement of significant others enhanced the quality, impact and learning from the review. It provided the victim with a ‘voice’ and made them ‘real’:

… [mother of victim] had a whole memory book. She had photos of her daughter. She really brought her alive. (Professional 2, Wales)

This was seen to encourage reflective practice, for example regarding encouraging DVA disclosure and routine enquiry. Conversely, where there was no involvement, important contextual information and the victim's voice were lost.

Professionals discussed navigating tricky waters where relatives were unaware of hidden aspects of the victim's life. Other challenges included difficult family dynamics, family disagreement regarding participation, and geographical distances. Professionals described situations where panel members or the Chair did not sufficiently understand or respect the views of significant others, or the dynamic/complexity of the situation:

It was very interesting to challenge the panel views and saying, “But this is not a review on what you believe. I’m telling you what the family have stated […] They’re the people that lived 24/7 with the [family].” (Professional 2, Wales)

Friends were consistently perceived as likely to have greater awareness of DVA than relatives. It was argued that more work to involve wider networks such as employers, neighbours, and communities would be beneficial, particularly where there was little agency (or family) involvement.

Professionals considered that DHRs could help significant others manage their grief, particularly as criminal proceedings focus on the perpetrator. While families’ primary motivation for DHR involvement was to prevent future harm to others, the lack of support after DHR completion was highlighted as a potential concern. There was also the recognition that families may feel disappointed or frustrated where learning did not help prevent another domestic homicide:

[…] it could have been prevented and there were failings within the system, you would be really offended and upset to think, well twelve months or two years later exactly the same thing happened and there was no learning from the previous one. (Professional 5, England)

More positively, professionals mentioned that often relatives write to express their gratitude for the work undertaken or that being involved helps them raise awareness or funds for local DVA services.

#### 4.2.3 Children's presence

Professionals reported that children were less likely to be involved in DHRs as “the incidents that are recounted by family members are often very disturbing” (Professional 9, England). But children could be indirectly involved via relatives or a social worker using a story/memory book or wishes and feelings activities through the individual management review process. In one case, support from a specialist victim service was offered where the child had given evidence during the trial. There was concern that reports should consider any future impact on children including their reading of the DHR as an adult.

### 4.3 NEGOTIATING ORGANISATIONAL BLAME TO FOSTER LEARNING

#### 4.3.1 The importance of learning events

Most professionals cited the learning event as a key mechanism to embed learning, share good practice and take recommendations forward. This was described as “very powerful” (Professional 2, Wales), especially as the need to improve practice without “finger pointing” was acknowledged (Professional 1, England). Practitioners need to be reassured that the events are not about blame:

Only one person, in most cases, killed that individual and that is the perpetrator and not all the rest of you sat round the table. You did not kill them. You did not set out to kill them and therefore you do not take any blame for it. (Professional 9, England)

Professionals with longstanding experience of DHRs commented on the way things have improved in the learning events. Growing trust in the learning events “has helped enormously to actually […] have that confidence to share and be quite candid” (Professional 9, England).

These events are difficult for both those moderating it and those who are the ‘centre of attention’ because of their failings. Well‐managed learning events are crucial as there are difficult conversations to navigate and no agency wants to be shamed in public:

But there is definitely still a reservation around the room from agencies that they do not want to be the ones with all the recommendations. It needs to be balanced. There is still some feeling of that being tied up with blame. (Professional 1, England)

#### 4.3.2 The blame culture

Professionals were cognisant that DHRs are required by statute and oversight sits with the Home Office. As a government department mainly responsible for law and order, its role is often associated with legality, accountability and blame. It was therefore thought that organisations might be more guarded and mistrust the process:

[…] that came from police officers too who quite often would not be happy to talk to panels about it because they just thought they would be thrown under a bus and blamed. (Professional 5, Wales)

Professionals stress that, in a culture of blame, it is difficult for those involved in the DHR process to be open, challenging of self and others, challenging organisational practice and managing different hats and roles:

Because sometimes you are representing the [name of agency] and you have got a corporate role but you have also got a duty of candour. (Professional 8, Wales)

There may also be a shifting of responsibility ‘as some partners [are] very defensive and they try to shift the blame’ (Professional 8, Wales). The corporate role creates a tension for representatives from organisations between what their organisation will allow them to say, the shifting of blame and the Chair's final report.

### 4.4 ACTIONING AND AUDITING RECOMMENDATIONS

#### 4.4.1 Implementation challenges

As DHRs can be lengthy, professionals explained that actioning recommendations frequently occurred during the DHR process, to ensure learning was relevant and timely. For effective implementation, participants stated that action plans should be (organisation) specific, tangible, achievable and realistic. Broad or vague recommendations such as ‘improved information‐sharing’ disperse responsibility and cause difficulties around monitoring implementation:

Well, how and when would that have been instigated and whose responsibility would it have been to share that information? (Professional 8, England)

Barriers to implementation included the Covid‐19 pandemic, austerity measures, commissioning arrangements, data protection, or referral pathways. For example, delivery of widespread training is time and resource intensive especially if these are embedded to account for changes in practice, legislation, and staff turnover. The lack of legal accountability or national strategic oversight leaves panels with insufficient power to ensure actions are undertaken, thus undermining the potential impact of DHRs:

If the learning from the review isn’t properly shared, understood, implemented, and creates change, then you have got to question what is the value in doing it? […] Who in the system is actually holding anybody to account for failures to implement recommendations from the last five homicide reviews? (Professional 5, England)

Professionals wanted improved action from senior leaders across organisations at local and national level to ensure learning and recommendations are actioned. DHRs often made national‐level recommendations, but local areas do not hold any authority regarding subsequent action.

#### 4.4.2 Impact of recommendations

Overall, the resultant learning and actions were valued but questions remained around measuring and evidencing changes generated by DHRs. To assist with measuring impact, it was suggested that consistency between action plans should be improved. Participants also highlighted that commissioners did not necessarily sit on DHR panels thus missing opportunities to commission services based on DHR recommendations.

A lack of long‐term oversight was mentioned as affecting the impact of DHRs:

It feels as though when reviews reach that publication phase they could fall off the edge of a cliff then because there’s not the governance or the scrutiny over what happens with the implementation of the learning as much as applying the process right up until that publication point. (Professional 2, Wales)

Professionals suggested that a DHR should be a continual process of ‘evolving practice’ rather than having a beginning and an end. This would include auditing, monitoring and evaluating recommendations. Accountability was considered important but there remained questions about who should do that and how it could be meaningful given resource constraints.

The Home Office was seen as central to facilitating, disseminating learning and monitoring change but this had not been forthcoming:

[…] where is this learning going to go now? We are at the Home Office, we have got this learning, are we going to send it to every single community safety partnership in England and Wales? Yes, we should. (Professional 5, Wales)

To facilitate co‐ordinated thematic learning and avoid duplication across time and place, participants suggested a national library or repository with search and analysis capability.

## 5 DISCUSSION

The discussion draws on key literature related to the objectives of the interviews capturing professionals’ experience of and views about the DHR process; the role of family and friends; implementation of recommendations; and key areas for improving practice.

In England and Wales, DHRs have been a statutory requirement since 2011, their conduct, scope, and remit being stipulated by the UK Government's Home Office. Given this centralised approach, it is perhaps surprising that our findings illustrate the variable quality of Chairs, the limited oversight of the learning or impact generated by DHRs.

DHRs are a complex process where the potential for learning can be hampered due to the defensiveness of panel members (and organisations involved) and the quality of the Chair. Although the emphasis of DHRs is on learning rather than blaming, it does not prevent professionals from fearing individual/professional/organisational blame. Websdale et al. (1999) stress the importance of moving away from a culture of blame to ‘creating a culture of safety in order to review domestic violence deaths effectively, honestly, and openly’ (1999:71). Defensiveness can be attributed to several factors: the tension between a panel member's corporate hat and organisation permission to speak versus the duty of candour; fear that publicly discussing organisational shortcomings may have personal repercussions; and the potential for shifting responsibility on to other organisations without exploring one's own organisation's practices—all of which may impede an honest reflection of potential improvements. Albright et al. (2013) recommend the adoption of an ethical code for conducting DHRs to mediate such tensions.

The role of the Chair was perceived as central to DHRs, yet many participants were critical of the quality of some Chairs. Chairs are required to be independent (Home Office, 2012) and democratising the DHR process to enhance its outcome includes three key factors: (a) utilising the knowledge and expertise of panel members as they are cognisant of the local context; (b) ensuring that relevant third sector/non‐governmental organisations are included in the panel; and (c) meaningful involvement with family members/victim's personal networks.

The need for appropriate training, codes of practice and quality assurance and a stringent recruitment process is highlighted by many participants. Rowlands (2020)’s international review recommends the development of a competencies framework for DHR Chairs and report authors, induction, training and a best practice network. Decisions about whether to proceed with a DHR are made collectively, but these decision‐making fora are unlikely to engage with third sector organisations who may have had contact with the victim or perpetrator (or families), thus potentially missing opportunities for improving practice.

The Home Office (2016): 17 DHR guidance notes that the quality and accuracy of the review is ‘likely to be significantly enhanced by family, friends and wider community involvement’ and they should therefore be treated as a key stakeholder, consistent with our findings here. Family involvement was believed to assist with grieving, humanise the victim and support prevention. However, the process for meaningful involvement is complex. Mullane (2017) indicates that where family involvement is not handled properly it can result in re‐traumatisation, highlighting the importance of specialist advocacy. Reasons for non‐involvement have been highlighted in earlier studies (Sharps‐Jeff & Kelly, 2016) and include the timing of the contact, concerns around re‐traumatisation and family dynamics. This might be overcome by offering greater flexibility around how and when families contribute (Mullane, 2017).

There is currently little information regarding the involvement of survivor/victim's personal networks (i.e. friends, neighbours, colleagues, community members) within the DHR process. This reflects the absence of provision aimed at informal supporters more generally (Gregory et al., 2016). Stanley et al. (2018) also found that children are rarely invited to contribute to the DHR process, despite an emphasis on the importance of hearing children's voices within the guidance.

According to Bugeja et al. (2013), only two jurisdictions globally mandate DHR panels to track recommendations. As our study illustrates, the implementation and evaluation of recommendations into concrete and evidence‐based actions to improve policy and practice responses to DVA is often elusive. Despite this, participants highlighted some good practice. However, their desire for increased Home Office involvement was clear, specifically harnessing the learning from DHRs across time and geography. The HM Government (2021) Domestic Abuse Act establishes the Domestic Abuse Commissioner as a statutory office that provides leadership on DVA nationally. This may assist the much‐needed centralised focus to ensure the investment in DHRs maximises learning and supports the monitoring and implementation of recommendations.

Lack of resources at both central and local level was reported as a key barrier. Austerity measures over the last decade have seen increasing thresholds and continuing cuts to statutory and third sector service provision in the UK and internationally (Barter et al., 2018; Sanders‐McDonagh & Neville, 2017; Sanders‐McDonagh et al., 2016; Sheehy, 2017; Warwick‐Booth & Cross, 2020), curtailing their ability to initiate or sustain systemic change.

## 6 LIMITATIONS

The findings are limited by a convenience sample of participants from two regions in England and Wales. Despite national Home Office guidance on the conduct of DHRs, there are variations in the way these are commissioned and conducted. The aim of this paper was not to compare global DHR practice, however, a literature review in this area (Rowlands, 2020) highlights similar issues regarding the need for quality assurance and a competencies framework for Chairs/report authors and DHR panel members. While some of the findings are transferable to other regions in the UK and internationally, there is a need for more follow‐up qualitative enquires to strengthen the evidence and enhance our understanding of individual, local, regional and national experiences of DHRs.

## 7 CONCLUSION

There is evidence of practice improvement since the introduction of DHRs in England and Wales in 2011, but also of reoccurring failures, especially linked to actioning recommendations and sustaining long‐term change.

Best practice involves a DHR process that is safe, transparent, evidence‐based and learning‐focused. This is highly dependent on a committed, objective, skillful and experienced Chair. Agreeing on and actioning the learning is a delicate process, challenging prejudices, the dominance of statutory organisations, governance rules and fear to speak out. Promoting the input of survivor family/networks and professionals on an equal footing is central. Maintaining transparency, ensuring strong leadership and quality assurance process are also key. Only through a successful, balanced collaboration with key stakeholders involved in the review can realistic recommendations be translated into practice.

There is a call for more regulation by and feedback from the Home Office. Raising awareness about recommendations that influenced change would enable more meaningful engagement in the DHR process from all parties. However, systemic change cannot be achieved without central government funding and commitment.

The terminology, methodology and legal base for DHR reviews need careful consideration if the focus on learning is to supersede that of apportioning blame. The way the review is conducted, in the lines of an enquiry or investigation into potential organisational failure, might encourage a defensive stance among professionals, to manage reputational risk. Organisational learning cannot be achieved without accepting organisational responsibility, which could be perceived as blame. Careful management enabling a safe open environment for all stakeholders is crucial to promote learning and help to develop good (if not best) practice in this area – for all involved.

## CONFLICT OF INTEREST

Haines‐Delmont, Bracell and Chantler have no conflict of interest to declare.

## Data Availability

The data that support the findings will be openly available in UK Data Service: https://ukdataservice.ac.uk/.
